# Spectacles for Dental Work

**Published:** 1900-03-15

**Authors:** W. Booth Pearsall


### SPECTACLES FOR DENTAL WORK.

Professor Laudolt's convex prismatic spectacles supply a want acutely felt by many dentists. The watchmaker's lens is inconvenient, and as it assists the sight of one eye only, loses the stereoscopic effect obtained by the use of both eyes. Strong convex glasses enlarge the object, but cause eyestrain from the effort necessary to convergence. In Professor Laudolt's spectacles a convex surface is ground on the surface of each of a pair of prisms, which are set in a spectacle frame. The convex surfaces focus, and as a prism refracts light toward its base accommodation and convergence are simultaneously relieved; the object is brought near the face and seen comfortably.—*W. Booth Pearsall, British Dental Journal.*

